# Normative Data for 111 Compound Remote Associates Test Problems in Romanian

**DOI:** 10.3389/fpsyg.2019.01859

**Published:** 2019-09-04

**Authors:** Ana-Maria Olteţeanu, Mihaela Taranu, Thea Ionescu

**Affiliations:** ^1^Department of Informatics and Mathematics, Freie Universität Berlin, Berlin, Germany; ^2^School of Psychology, University of Plymouth, Plymouth, United Kingdom; ^3^Department of Psychology, Babeş-Bolyai University, Cluj-Napoca, Romania

**Keywords:** creativity tests, remote associates test, normative data, creative cognition, Romanian

## Abstract

The Remote Associates Test (RAT) is a classical creativity test developed by Mednick and Mednick in 1967. RAT problems and their norms so far exist only in a few languages, including English, Dutch, Japanese and Italian. In this paper, we describe our process of constructing a set of Remote Associates Test problem in Romanian. 63 native speaking Romanian participants have solved this set. The set of items shows high internal consistency. Normative data pertaining to each problem is provided, together with a description of RAT problems peculiarities in Romanian.

## 1. Introduction

The Remote Associates Test is a widely used creativity test (Ansburg and Hill, 2003; Ward et al., 2008; Cai et al., 2009; Cunningham et al., 2009) by Mednick and Mednick (1971). A RAT problem consists of the following: three words are given to the participant, like Safety, Cushion, Point; the participant is asked to come up with a fourth word, which relates to all of the three given words. For example, for the problem above, Pin would be a good answer, because of the compounds Safety Pin, Pin Cushion, AND Pinpoint, which relate each of the three given words to the answer.

Associative abilities are considered an important factor in creativity (Benedek et al., 2012). Various versions of the RAT exist—compound (Bowden and Jung-Beeman, 2003), functional (Worthen and Clark, 1971; Olteţeanu et al., 2019), and visual (Olteţeanu et al., 2015). The compound RAT is so far the most widely used.

Normative data exists for the compound Remote Associates Test in a small set of languages: English (Bowden and Jung-Beeman, 2003); Italian (Salvi et al., 2016); Dutch (Chermahini et al., 2012); Japanese (Baba, 1982), Chinese (Shen et al., 2016), and Polish (Sobków et al., 2016).

In order to measure associative ability, the language in which the test is given is important; native speakers are much more likely to know the expressions and compound words in their language, thus to have the knowledge required to solve the test. The associative ability process can thus be measured reliably when the test is administered in the native language of the speaker.

In previous work, Remote Associates items have been both solved computationally (Olteţeanu and Falomir, 2015) and also created computationally in American English (Olteţeanu et al., 2017). While a computational approach to item creation could also work for other languages, the manual construction of a RAT in that language is generally indicated initially, in order to first understand the peculiarities of creating the test in that language. In order for researchers to be able to use the RAT to assess native Romanian speakers, this paper focuses on the construction and norming of a variant of the RAT in Romanian. The authors, all native Romanian speakers, have collaboratively created the items. The rest of the paper is organized as follows: the process of constructing the queries is described in section 2. The process of norming the queries, together with descriptive statistics on the results obtained, are provided in section 3. A discussion on the specific difficulties in creating RAT queries in Romanian is presented in section 4. The last section presents future work and conclusions, followed by an Appendix containing the normative data on each of the question items, and their partial translation to English.

## 2. Constructing Queries

The query construction process was done in four steps. These are:

1. Direct translation attempt;
2. Adaptation of items;
3. Creation of new items (including check of expressions);
4. Initial item evaluation and clean-up.

In the rest of this section we will present our process, for the benefit of researchers that are native speakers of other languages—they might be able to use this process to create RAT repositories in their own language.

### 2.1. Direct Translation Attempt

We initiated the procedure of constructing queries by exploring the English and Italian stimuli provided in the normative data of Bowden and Jung-Beeman (2003) and Salvi et al. (2016). As the co-authors were familiar with both of these languages, we first explored whether some of these items would translate in RAT items in Romanian. In the following we will exemplify how some queries may or may not be directly translatable.

Let us take as an example a formal query *w*_1_, *w*_2_, and *w*_3_, where *ans*_*x*_ is considered a correct answer. For this query to be possible in language *L*_1_, at least three expressions or compound words must exist in that language:

- (*w*_1_, *ans*_*x*_) or (*ans*_*x*_, *w*_1_);
- (*w*_2_, *ans*_*x*_) or (*ans*_*x*_, *w*_2_) and
- (*w*_3_, *ans*_*x*_) or (*ans*_*x*_, *w*_3_).

For this query to be directly translatable from *L*_1_ to another language *L*_2_, these expressions or compound words, either in the same or reverse order, must also exist in *L*_2_ (though they need not have the same meaning in *L*_2_). For example, the query Casella Prioritaria Elettronica, answer Posta, can be directly translated from Italian to the English query Box Priority Electronic, answer Mail, because all three compounds Mail Box, Priority Mail, and Electronic Mail are valid. The answer Posta could also be translated more directly to the word Post, however using this word as a potential answer for the RAT does not work very well, because Electronic Post is not a compound used in English.

Most queries are not directly translatable, because one of the expressions does not exist in the second language. For example, for the above mentioned query, a direct translation in Romanian would involve the words Căsuţă (box), Prioritară (with priority), and Electronică (electronic) with the answer Poştă (post). However, the answer word has to be an adjective in order fit with Căsuţă (i.e., the correct form is Căsuţă Poştală and not Căsuţă Poştă). On the other hand, changing the word Poştă to Poştală makes the other two compounds invalid. If two out of the three queries existed, we proceeded to then adapt the query to the new language.

### 2.2. Adaptation of Items

Adaptation of an existing query to a new language happened as follows. If when attempting to translate a RAT query from *L*_1_ to *L*_2_ only items (i) (*w*_1_, *ans*_*x*_) or (*ans*_*x*_, *w*_1_) and (ii) (*w*_2_, *ans*_*x*_) or (*ans*_*x*_, *w*_2_) were valid expressions or compound nouns in *L*_2_, but (*w*_3_, *ans*_*x*_) or (*ans*_*x*_, *w*_3_) did not exist, we replaced *w*_3_ with a new word, which formed a valid expression or compound noun with *ans*_*x*_ in *L*_2_.

For example, in Salvi's (Salvi et al., 2016) set of items, we encountered the item: Bianca Credito Identità, answer Carta. In Romanian, the expressions formed with the answer word by the second and third terms exist: Carte de Credit (credit card) and carte de identitate (identity card). However, for Carta Bianca (roughly meaning a free pass), Romanian uses the French Carte Blanche. We thus had to replace *w*_1_ Bianca—in Romanian Albă with Sănătate (health), which forms a valid expression with the answer Card de Sănătate (health card).

The RAT item thus obtained after adaptation was Sănătate, Credit, Identitate, answer Card.

### 2.3. Creation of New Items

In many cases, not just one of the three terms, but two of the three terms did not form a good compound. However, one of the query items and the answer could still be taken as a seed for creating new queries. We developed this technique when observing the similarity of one query word and the answer word between the two sets of items in English and Italian. For example, in Bowden's set of queries, Cottage, Swiss, Cake, answer Cheese appears. In Salvi's set of queries, we encounter item Capra, Svizzero, Buchi, answer Formaggio. The second word of the query and the answer form a compound (Swiss Cheese—Formaggio Svizzero) which is common to the two queries. However, the two other queries in Italian translate roughly as *goat cheese* and *cheese with holes*. In Romanian we made the query: Capră (goat), Topită (melted), Burduf (bellows), answer: Brânză (cheese).

While creating new RAT items, we sometimes used one query word-answer word pair, or just a query word or an answer word as a seed. That is to say, we formed a completely new query using that seed word or compound, upon finding a word we considered would have suitable other compounds in Romanian. During query creation, we used an online Romanian dictionary^1^ to check whether other compounds existed for a particular word, and whether some compounds where as usual as we thought them to be.

### 2.4. Initial Item Evaluation and Clean Up

After the steps above, a list of 198 RAT items was established. The authors and five Romanian student volunteers rated all the items on a 1–5 Likert scale (1 meant an excellent item), and made notes on items which did not seem suitable. Thus eight ratings were obtained for each of the 198 items. The top rated items where kept, and some weaker items improved based on suggestions from the various raters.

The resulting set of items was then cleaned of query word repetitions. This was performed as not to bias participants to associate a particular query word with a particular compound, because of having encountered that query word earlier during the solving in a different potential compound. Where two queries both contained a particular query word, we kept the stronger rated query. After this process, a set of 111 RAT queries in Romanian was obtained.

## 3. Norming Queries

The 111 queries thus created where given to participants in order to evaluate and norm.

### 3.1. Method

Participants where invited to take part in the norming via the online platform CrowdFlower^2^, a platform similar to Mechanical Turk. The call invited native level Romanian speakers alone, and restricted IP addresses of potential participants to Romania. Volunteer students from Babeş-Bolyai University, Cluj, Romania also participated. In accordance with local legislation, the study was exempt from an ethics committee approval because it only involved the gathering of normative data on a task that posed no danger to human participants. Participants approved to having their data used in anonymised form for scientific purposes as part of continuing the online data gathering process.

### 3.2. Participants

There were 63 participants (31 females, 32 males), aged between 18 and 70 years (3 under 20; 27 between 20 and 30 years; 19 between 30 and 40 years; 9 between 40 and 50 years; 4 between 50 and 60 years of age; and 1 between 60 and 70 years of age). With regard to their educational level, 9 of them graduated high-school, 11 were university students, 32 had a bachelor degree, 3 were graduate students, and 8 had a post graduate degree.

### 3.3. Procedure

Each participant first provided personal data and consent for the use of their anonymised data. The task was then explained, using two examples for which answer words were provided. These examples where:

- Example query 1: Boabe (Grains), Bătută (Mashed),Verde (Green). Answer: Fasole (Beans);
- Example query 2: Păr (Hair), Haine (Clothes), Pantofi (Shoes). Answer: Perie (Brush).

The examples where explained by showcasing the compounds in which each answer word was associated with each of the three query words: Fasole Boabe, Fasole Bătută and Fasole Verde for example 1, and Perie de Păr, Perie de Haine and Perie de Pantofi for example 2, respectively.

Four training queries where then provided, to familiarize the participant with the task. After the participant answered each of the training queries, the expected answer for the query was provided. It was explained that *word x can be associated with each of the three given words:* and then the three required compounds where provided. The four training queries were the following:

- Training item 1: Expres (Express), Regional (Regional), Călători (Passengers). Answer: Tren (Train);
- Training item 2: Ferată (Rail), Scăpare (Exit), Lungă (Long). Answer: Cale (Road);
- Training item 3: Scafandru (Scuba-driver), Naţional (National), Baie (Swim). Answer: Costum (Suit);
- Training item 4: Capră (Sheep), Topită (Melted), Burduf (Sacked). Answer: Brânză (Cheese).

Training queries were also used to assess whether each participant understood the task.

After the training queries, the 111 queries for which normative data was sought were then given in randomized order. We measured accuracy and response times. Response times were recorded using jsPsych, a JavaScript library for running behavioral experiments in a web browser^3^. The trials were administered without any breaks.

### 3.4. Results

The overall average accuracy to complete all the items was *M* = 0.54 (*SD* = 0.43). Response times (RT) were calculated in seconds. RTs were recorded for both correctly answered and incorrectly answered items. The average reaction time for the items solved correctly was *M* = 15.37 (*SD* = 10.53). The average reaction time for solving all the items (including the incorrect responses) was *M* = 22.41 (*SD* = 22.39). Descriptive data on accuracy and response times, together with confidence intervals, is shown in Table 1.

**Table 1:** Descriptive statistics on accuracy and response times, *n* = 63.

	**Accuracy**	**RT correct**	**RT all**
Average (SD)	0.54 (0.43)	15.37 (10.53)	22.41 (22.39)
SE	0.05	2.36	2.99
95% CI. LB	0.43	10.74	16.55
95% CI. UB	0.64	19.99	28.27

**Figure 1:**
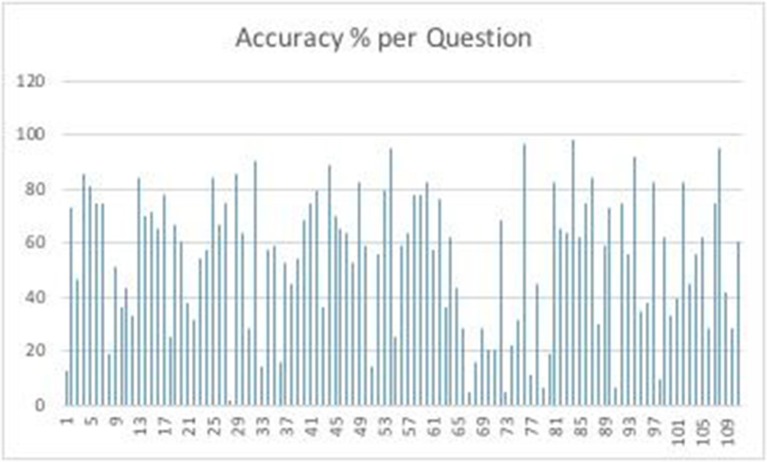
Accuracy per question.

The accuracy and response times to each of the query items are shown in the Appendix. Accuracy per question can be seen in Figure 1.

The easiest query was item number 84: GURĂ, MINERALĂ, IZVOR, answer APĂ (Mouth, Sparkling, Spring, answer Water), with an average accuracy of 0.98. The hardest query was item number 28: FÂN, RÂU, MUNCĂ, answer BRAŢ (Hay, River Work, answer Hand/Arm) with an average accuracy of 0.02. Overall, the fastest query solved was item number 76: LUMINĂ, BISECT, CALENDARISTIC, answer AN (Light, Leap, Calendar, answer Year) with an average reaction time of 7.25 s. The slowest solved query was item number 98: ADEVĂR, MĂR, LUMINĂ, answer SÂMBURE (Truth, Apple, Light, answer Seed) with an average reaction time of 42.81 s. Item number 76 was also the fastest correct query solved (average reaction time of 7.11 s). The slowest correct answered query was item number 91: LOC, DREPT, FACTOR, answer COMUN (Place, Right, Factor, answer Common).

The scale reliability of the newly created set of items was measured using Cronbach's alpha as an internal consistency measure. Cronbach's alpha on Accuracy was 0.93; Cronbach's alpha on reponse times (on both correct and incorrect items) was 0.97.

Seventy three percent of the participants had ages between 20 and 40, 22% were aged between 40 and 70 and 4% of the participants were younger than 20 years. There were no age effects on accuracy, *F*_(2, 60)_ = 0.048, *p* = 0.953, nor on reaction times for correct responses, *F*_(2, 60)_ = 0.998, *p* = 0.374.

Gender differences were observed on the accuracy of responses, females being more accurate than males, *F*_(1, 61)_ = 5.225, *p* = 0.026. There were no gender effects on the reaction times of the correct responses, *F*_(1, 61)_ = 0.359, *p* = 0.551. Although the number of participants is relatively low this result is very interesting as it shows some cultural differences in the effects of gender on RAT responses.

No significant correlation between self-assessed creativity and accuracy or response times was observed.

At the request of one of our reviewers, we also provided a partial translation of the query items in English. The translations are followed by notes. The translations are of course not meant to be RAT items, but rather help give insight into language differences.

## 4. Discussion

A set of 111 compound Remote Associates Test problems was created in Romanian. The high Cronbach alpha of the answers to these items, measured for both accuracy and response times, shows the scale has high internal consistency. This validates our approach to item creation.

On a general note, the intent of this manuscript is to showcase an approach to RAT items creation for languages in which the RAT does not exist. We believe that our general process of item creation, described in section 2, is applicable to most other languages too. This process can be summarized as starting from existing sets of RAT items in languages the set creators understand, followed by: (1) a direct translation attempt from the source language(s); (2) an adaptation of the items that cannot be fully translated, using the target language's expressions; (3) creation of new items in the target language and (4) initial item evaluation and clean-up. We would generally like to encourage other researchers to create sets of RAT items in their native languages, as this will provide more culturally diverse studies of creativity, and cross-cultural comparison. Such cross-cultural comparisons can further support cross-modality studies of the associative process (Olteţeanu et al., 2015; Toivainen et al., 2019). A bigger picture of the role associative processes play in creativity can then be obtained.

After the description of this approach, we hope computational approaches can be implemented to generate items in different languages, like the one proposed by Olteţeanu et al. (2017), Olteţeanu et al. (2019), and Olteţeanu et al. (2018), offering more parameter control (Olteţeanu and Schultheis, 2017). However, we would like to emphasize that computational creation acts best when based on direct observations of expert researchers regarding peculiarities of that language, as language differences will involve tweakings of the general process of item creation; such observations can only be made via studying or creating and validating an initial set of RAT items in that language.

One peculiarity of the Romanian language is that compounds often use prepositions (like Perie de Păr for Hair Brush). Moreover, there are cases in which only some of the compounds need presposition while others do not. For example, for the query Pălărie (Hat), Poştală (Mail), Carton (Cardboard), answer Cutie (Box), only Cutie de Pălărie (Hat Box) and Cutie de Carton (Bardboard Box) use prepositions while Cutie Poştală (Mail Box) does not. In addition, sometimes different prepositions are used in the same query: Rază (Ray), Floare (flower), Rupt (Broken), answer Soare (Sun); the compounds are Rază de Soare (sunbeam), Floarea-Soarelui (sun-flower), and Rupt din Soare (broken from the sun—Romanian idiom aimed to represent something that is particularly beautiful).

Another peculiarity is that Romanian nouns with determiners (e.g., Cartea vs. Carte, translated to The Book vs. Book; see Floarea-Soarelui above) cannot be used in the same format as answers because they are not appropriate in this format to other compounds. Again, respondents were not instructed when or whether to use determiners: for example, they had to come up with the answer Soare (sun), but they had to think that in combination with Floare (flower) both must change to nouns with determiners, Floarea-Soarelui (literally, the flower of the sun in Romanian).

These two features of compounds in Romanian language also made adapting the original queries difficult.

The easiest and fastest questions are not the same. This may be due to overt processes of checking the intuited answer fits with all query items increasing Accuracy for some participants and queries. It may also be due to the fact that easier queries to answer (queries which are answered correctly with higher frequency) include RT contributions from relatively slower solvers, while queries that are harder do not include such RTs because slower solvers may be unable to accurately solve these queries.

An interesting question which one of our reviewers brought forth was what makes an answer a correct answer. For the context of this paper, we have assessed correctness of the answer word to represent the fact that a relationship existed between the answer word and each of the query words. The uniqueness of the correct response is, however, a different matter. As one of the authors has pointed out in Olteţeanu and Falomir (2015), the computational solver they built for the solving of the RAT in English showed that other answer were possible and correct, besides the answers rated as correct in the norms of Bowden and Jung-Beeman (2003). Thus while the Romanian queries manually created here did not evoke any oher answers to the authors, a more complete exploration of whether other correct answers are possible could only be done computationally, as part of future work.

## 5. Future Work and Conclusion

In this paper, a set of 111 compound Remote Associates Test problems was developed in Romanian. This set of queries was normed using Romanian participants. These queries can now be used in experiments which involve creativity measurements for Romanian participants. A formal description of the query creation process was also provided, so that computational forms of this process can be implemented in the future.

As future work, we plan to:

- prototype a computational form of constructing Romanian RAT queries, based on our process description and on processes of computational query construction already deployed in English (Olteţeanu and Falomir, 2015);
- explore queries which are common across multiple languages with bilingual participants.

## Ethics Statement

The study was exempt because it only involved the gathering of normative data on a task that posed no danger to human participants.

## Author Contributions

A-MO, MT, and TI contributed to design of queries in Romanian, data gathering, and data analysis.

### Conflict of Interest Statement

The authors declare that the research was conducted in the absence of any commercial or financial relationships that could be construed as a potential conflict of interest.

## Footnotes

^1^https://dexonline.ro/

^2^https://www.crowdflower.com/

^3^https://www.jspsych.org/
