# Supplementary material for: Normative Data for 111 Compound Remote Associates Test Problems in Romanian
Source: Front Psychol. 2019 Sep 4;10:1859. doi: 10.3389/fpsyg.2019.01859 (PMC6737282; doi:10.3389/fpsyg.2019.01859)
Supplement: Supplementary file 1 [file Data_Sheet_1.pdf]

Appendix 1 - Accuracy and Response Times per Query

No.	Word 1	Word 2	Word 3	Answer	Accuracy %	Accuracy mean (SD)	RT correct mean (SD)	RT all mean (SD)	RT incorrect
1	Francez	Vânătoare	Ciocolată	Corn	12.7	0.13 (0.34)	17.96 (9.17)	39.52 (30.03)	41.53 (30.55)
2	Principală	Măturător	Lumină	Stradă	73.02	0.73 (0.45)	15.08 (13.23)	23.7 (26.16)	37.01 (35.2)
3	Companie	Vânătoare	Nevăzători	Câine	46.03	0.46 (0.5)	14.58 (11.82)	26.32 (25.51)	35.92 (29.8)
4	Munte	Lance	Ascuţit	Vârf	85.71	0.86 (0.35)	9.93 (7.76)	12.07 (11.34)	24.59 (20.9)
5	Rază	Floare	Rupt	Soare	80.95	0.81 (0.4)	14.8 (10.96)	18.91 (16.77)	29.69 (23.45)
6	Întrebare	Circulaţie	Zodiacal	Semn	74.6	0.75 (0.44)	9.14 (5.73)	15.9 (18.66)	32.75 (27.89)
7	Cerneală	Rezervor	Peniţă	Stilou	74.6	0.75 (0.44)	15.57 (11.03)	22.28 (20.95)	38.35 (28.83)
8	Sârmă	Vârfuri	Înainte	Mers	19.05	0.19 (0.4)	23.11 (15.38)	27.51 (28.9)	28.32 (30.83)
9	Corali	Trecere	Socială	Barieră	50.79	0.51 (0.5)	17.4 (14.73)	25.3 (24.3)	29.78 (20.32)
10	Pretext	Acoperire	Egida	Sub	36.51	0.37 (0.49)	21.54 (22.79)	24.82 (25.52)	26.8 (27.15)
11	Replică	Ploaie	Băutură	Acidă	42.86	0.43 (0.5)	15.23 (8.57)	20.53 (16.23)	24.16 (19.19)
12	Şoareci	Întrebare	Automobil	Capcană	33.33	0.33 (0.48)	26.43 (19.74)	33.67 (27.71)	36.8 (30.23)
13	Urgenţă	Telefonic	Curte	Apel	84.13	0.84 (0.37)	12.77 (7.02)	15.03 (12.05)	19.04 (12.12)
14	Vizual	Tragere	Semantic	Câmp	69.84	0.7 (0.46)	12.46 (8.66)	15.27 (12.68)	21.44 (17.86)
15	Mandat	Oficiu	Timbru	Poştal	71.43	0.71 (0.46)	9.91 (6.31)	14.64 (27.09)	25.55 (47.1)
16	Scris	Cusut	Ulei	Maşină	65.08	0.65 (0.48)	20 (17.83)	25.99 (23.88)	35.14 (28.85)
17	Noapte	Rasă	Zăpadă	Albă	77.78	0.78 (0.42)	14.91 (9.25)	21.83 (32.98)	43.04 (63.71)
18	Zâmbet	Cântă	Document	Fals	25.4	0.25 (0.44)	13.75 (6.94)	35.61 (39.49)	42.89 (43.13)
19	Navală	Sportivă	Date	Bază	66.67	0.67 (0.48)	11.62 (10.73)	15.15 (15.34)	23.82 (21.15)
20	Faţă	Tenis	Moleculară	Masă	60.32	0.6 (0.49)	14.05 (11.34)	27.2 (38.83)	41.22 (52.35)
21	Franceză	Poziţie	Cuvânt	Cheie	38.1	0.38 (0.49)	17.56 (13.09)	30.64 (33.1)	35.11 (36.37)
22	Perete	Ultimul	Electronic	Ceas	31.75	0.32 (0.47)	23.07 (25.15)	30.83 (27.55)	34.2 (28.17)
23	Damă	Pantaloni	Transmisie	Curea	53.97	0.54 (0.5)	14.57 (8.48)	25.63 (21.8)	35.29 (24.75)
24	Tun	Vită	Vie	Carne	57.14	0.57 (0.5)	14.53 (13.03)	25.5(30.28)	36.72 (37.86)
25	Pauză	Boabe	Filtru	Cafea	84.13	0.84 (0.37)	9.96 (4.69)	11.14 (6.25)	18.82 (9.97)
26	Verde	Orientală	Fructe	Salată	66.67	0.67 (0.48)	9.89 (7.95)	16.08 (20.84)	28.6 (31.66)
27	Perete	Miez	Cocos	Nucă	74.6	0.75 (0.44)	13.41 (11.67)	17.3 (14.62)	28.39 (16.88)

Continued on next page

Continued from previous page

No.	Word 1	Word 2	Word 3	Answer	Accuracy %	Accuracy mean (SD)	RT correct mean (SD)	RT all mean (SD)	RT incorrect
28	Fân	Râu	Muncă	Braţ	1.59	0.02 (0.13)	37.08 (0)	37.03 (25.13)	37.02 (25.43)
29	Dublă	Video	Obscură	Cameră	85.71	0.86 (0.35)	9.53 (7.97)	11.69 (12.18)	22.53 (22.36)
30	Natalităţii	Schimb	Bancară	Rată	63.49	0.63 (0.49)	13.17 (18.55)	16.55 (18.71)	22.16 (18.09)
31	Ultima	Actuală	Fixă	Oră	28.57	0.29 (0.46)	11.75 (6.1)	17.36 (15.73)	19.57 (17.67)
32	Rinichi	Filosofală	Seacă	Piatră	90.48	0.9 (0.3)	9.31 (6.68)	9.9 (8.74)	18.4 (19.22)
33	Pantofi	Dansatori	Fără	Pereche	14.29	0.14 (0.35)	27.29 (8.21)	32.23 (29.84)	32.84 (31.49)
34	Legitimă	Avocat	Cheltuieli	Apărare	57.14	0.57 (0.5)	13.77 (9.11)	18.94 (16.69)	24.67 (21)
35	Ajutor	Lucru	Dibace	Mână	58.73	0.59 (0.5)	17.36 (14.18)	21.08 (17.83)	23.37 (16.26)
36	Gata	Doi	Gheaţă	Bani	15.87	0.16 (0.37)	9.07 (2.99)	31.93 (38.8)	36.14 (40.89)
37	Ou	Pisică	Picături	Ochi	52.38	0.52 (0.5)	21.99 (18.37)	32.09 (27.3)	41.83 (31.07)
38	Cal	Muncă	Economică	Putere	44.44	0.44 (0.5)	17.29 (17.07)	26.91 (29.51)	34.5 (34.87)
39	Electronică	Prioritară	Romană	Postă	53.97	0.54 (0.5)	12.77 (7.82)	21.54 (23.33)	32.64 (31.06)
40	Iarbă	Păr	Telefon	Fir	68.25	0.68 (0.47)	9.64 (4.48)	15.88 (16.29)	29.17 (23.89)
41	Sănătate	Credit	Identitate	Card	74.6	0.75 (0.44)	8.39 (5.97)	11.87 (21.79)	21.16 (40.19)
42	Biliard	Chibrit	Pescuit	Băţ	79.37	0.79 (0.41)	10.6 (7.01)	17.51 (22.7)	32.42 (35.17)
43	Mac	Vedea	Fier	Roşu	36.51	0.37 (0.49)	21.64 (14.15)	32.44 (28.96)	35.53 (31.44)
44	Marinăresc	Gordian	Cravată	Nod	88.89	0.89 (0.32)	7.42 (3.83)	8.42 (7.56)	15.86 (19.4)
45	Mese	Bal	Aşteptare	Sala	69.84	0.7 (0.46)	12.43 (9.61)	15.77 (16.27)	23.95 (24.6)
46	Practic	Şaselea	Bun	Simţ	65.08	0.65 (0.48)	10.52 (6.56)	16.96 (13.64)	26.3 (14.13)
47	Interzis	Dublu	Figurat	Sens	63.49	0.63 (0.49)	10.48 (5.33)	14.06 (12.69)	19.49 (17.91)
48	Socru	Capul	Literă	Mare	52.38	0.52 (0.5)	21.45 (14.84)	29.52 (29.34)	36.66 (37.7)
49	Argint	Invitaţie	Alai	Nuntă	82.54	0.83 (0.38)	15.52 (13.32)	19.75 (20.57)	31.68 (31.06)
50	Astral	Diplomatic	Delict	Corp	58.73	0.59 (0.5)	11.83 (7.4)	19.8 (18.93)	29.21 (23.64)
51	Drum	Timp	Circuit	Scurt	14.29	0.14 (0.35)	14.95 (11.57)	28.74 (37.61)	30.63 (39.57)
52	Sânge	Turcească	Abur	Baie	55.56	0.56 (0.5)	9.37 (4.77)	16.28 (15.55)	26.38 (19.32)
53	Secret	Rutier	Civil	Cod	79.37	0.79 (0.41)	7.61 (3.54)	9.75 (7.33)	18.15 (12.15)
54	Rulantă	Adezivă	Magnetofon	Bandă	95.24	0.95 (0.21)	9.52 (8.73)	9.32 (8.66)	6.63 (6.84)
55	Ziuă	Botez	Familie	Nume	25.4	0.25 (0.44)	17.01 (10.96)	27.66 (28.84)	29.06 (29.34)
56	Oratorică	Culinară	Contemporană	Artă	58.73	0.59 (0.5)	11.05 (7.05)	16.66 (14.97)	24.41 (18.96)
57	Unghii	Pantofi	Glaciar	Lac	63.49	0.63 (0.49)	13.42 (11.58)	20.17 (17.84)	27.78 (21.41)

Continued on next page

Continued from previous page

No.	Word 1	Word 2	Word 3	Answer	Accuracy %	Accuracy mean (SD)	RT correct mean (SD)	RT all mean (SD)	RT incorrect
58	Ski	Biciclişti	Aterizare	Pistă	77.78	0.78 (0.42)	10.65 (8.23)	17.07 (19.57)	36.59 (28.54)
59	Uşa	Pantof	Cui	Toc	77.78	0.78 (0.42)	13.47 (10.17)	19.32 (22.45)	28.03 (22.3)
60	Zăcamânt	Nisipuri	Medalie	Aur	82.54	0.83 (0.38)	11.87 (7.42)	12.92 (8.63)	18.17 (11.37)
61	Corporală	Capitală	Crimă	Pedeapsă	57.14	0.57 (0.5)	16.85 (11.09)	23.45 (21.55)	30.94 (28.25)
62	Greşit	Primul	Dans	Pas	76.19	0.76 (0.43)	12.33 (12.28)	22.52 (28.57)	43.84 (39.88)
63	Miazăzi	Briză	Vorbă	Vânt	36.51	0.37 (0.49)	13.26 (6.1)	32.02 (32.84)	41.43 (36.08)
64	Electric	Cont	Literar	Curent	61.9	0.62 (0.49)	14.71 (6.33)	27.95 (24.26)	42.62 (28.62)
65	Harta	Turul	Capătul	Lumii	42.86	0.43 (0.5)	9.83 (4.24)	17.4 (15.97)	22.96 (18.96)
66	Piept	Pernă	Vrabie	Pui	28.57	0.29 (0.46)	25.36 (10.8)	32.85 (39.25)	34.49 (42.8)
67	Ţine	Proaspătă	Tăiată	Respiraţia	4.76	0.05 (0.21)	11.02 (1.37)	30.84 (32.15)	31.7 (32.58)
68	Producţie	Transport	Exprimare	Mijloc	15.87	0.16 (0.37)	13.84 (7.64)	28.32 (37.18)	31.04 (39.86)
69	Prima	Platonică	Adevărată	Dragoste	28.57	0.29 (0.46)	11.59 (5.02)	14.59 (16.98)	15.68 (19.59)
70	Gaz	Informaţii	Canal	Scurgere	20.63	0.21 (0.41)	17.38 (9.89)	33.54 (29.84)	36.29 (31.26)
71	Limba	Iubire	Linie	Maternă	20.63	0.21 (0.41)	18.15 (5.09)	31.63 (29.98)	34.01 (31.8)
72	Puşcă	Lapte	Stele	Praf	68.25	0.68 (0.47)	11.7 (8.13)	18 (16.95)	28.56 (31.8)
73	Scris	Lucru	Spălat	Mână	4.76	0.05 (0.21)	17.16 (10.97)	22.23 (22.18)	22.49 (22.63)
74	Maximă	Ideală	Centru	Greutate	22.22	0.22 (0.42)	26.54 (16.27)	30.91 (26.31)	31.97 (28.29)
75	Control	Şah	Fildeş	Turn	31.75	0.32 (0.47)	18.21 (19.06)	25.32 (25.87)	28.44 (27.98)
76	Lumină	Bisect	Calendaristic	An	96.83	0.97 (0.18)	7.11 (5.36)	7.25 (5.53)	10.61 (8.96)
77	Tele	Călătorie	Bord	Jurnal	11.11	0.11 (0.32)	17.1 (4.44)	29.86 (24.47)	31.14 (25.29)
78	Şarpe	Neagră	Ureche	Gaură	44.44	0.44 (0.5)	15.03 (17.09)	25.13 (29.86)	35.03 (35.96)
79	Agricolă	Generală	Minister	Cultură	6.35	0.06 (0.25)	15.52 (5.06)	33.88 (31.92)	34.92 (32.5)
80	Forţă	Noapte	Neagră	Cămaşă	19.05	0.19 (0.4)	21.23 (10.42)	32.79 (26.74)	34.51 (28.04)
81	Pescuit	Salvare	Pneumatică	Barcă	82.54	0.83 (0.38)	9.23 (5.02)	10.32 (7.83)	15.45 (14.8)
82	Rotile	Electric	Birou	Scaun	65.08	0.65 (0.48)	14.72 (11.92)	22.08 (22)	32.92 (28.67)
83	Lună	Albine	Mană	Miere	63.49	0.63 (0.49)	17.71 (13.77)	32.29 (42.89)	48.21 (57.29)
84	Gură	Minerală	Izvor	Apă	98.41	0.98 (0.13)	9.16 (7.9)	9.35 (8.24)	20.57 (20.41)
85	Tropicală	Protejată	Amazoniană	Pădure	61.9	0.62 (0.49)	12.06 (11.21)	12.69 (12.18)	14.27 (13.72)
86	Hoţi	Rulantă	Circulaţie	Bandă	74.6	0.75 (0.44)	11.17 (5.89)	12.12 (6.61)	14.78 (7.79)
87	Artificii	Armă	Lemne	Foc	84.13	0.84 (0.37)	9.42 (6.2)	11.04 (9.23)	14.24 (9.25)

Continued on next page

Continued from previous page

No.	Word 1	Word 2	Word 3	Answer	Accuracy %	Accuracy mean (SD)	RT correct mean (SD)	RT all mean (SD)	RT incorrect
88	Ac	Centură	Naţională	Siguranţă	30.16	0.3 (0.46)	21.28 (22.48)	35.81 (32.46)	40.94 (34.12)
89	Muzicală	Direcţie	Bijuterii	Cutie	58.73	0.59 (0.5)	13.37 (16.83)	19.79 (21.96)	28.22 (25.17)
90	Justiţie	Şcolii	Constituţională	Curtea	73.02	0.73 (0.45)	15.42 (10.72	18.36 (19.61)	26.3 (30.94)
91	Loc	Drept	Factor	Comun	6.35	0.06 (0.25)	56.96 (0)	37.14 (29.44)	36.69 (29.62)
92	Vizită	Bucate	Joc	Carte	74.6	0.75 (0.44)	13.37 (12.97)	21.6 (28.37)	42.11 (45.18)
93	Cont	Organe	Naţională	Bancă	55.56	0.56 (0.5)	17.25 (15.96)	28.32 (32.14)	40.83 (40.02)
94	Metil	Voroneţ	Cer	Albastru	92.06	0.92 (0.27)	11.02 (9.58)	12.13 (11.65)	13.1 (11.82)
95	Rufe	Vase	Clăbuci	Săpun	34.92	0.35 (0.48)	18.9 (13.93)	22.13 (27.99)	20.73 (28.85)
96	Comercial	Vânzare	Cosmic	Spaţiu	38.1	0.38 (0.49)	13.99 (6.33)	18.41 (16.11)	20.61 (18.92)
97	Dormit	Plastic	Gunoi	Sac	82.54	0.83 (0.38)	7.27 (5.19)	9.4 (10.36)	17.92 (19.39)
98	Adevăr	Măr	Lumină	Sâmbure	9.52	0.1 (0.3)	40.59 (46.72)	42.81 (39.47)	43 (39.38)
99	Spălat	Tuns	Cusut	Maşină	61.9	0.62 (0.49)	11.22 (8.3)	17.9 (17.89)	26.01 (22.69)
100	Categorie	Autobuz	Concert	Bilet	33.33	0.33 (0.48)	24.14 (30)	36.31 (36.12)	41.04 (37.76)
101	Proprietate	Viaţă	Întreprindere	Privată	39.68	0.4 (0.49)	14.71 (12.14)	18.21 (16.09)	20.57 (18.06)
102	Socială	Elevi	Muncitoare	Clasă	82.54	0.83 (0.38)	9.32 (4.25)	11.06 (8.53)	19.39 (16.35)
103	Geam	Inculpat	Drum	Închis	44.44	0.44 (0.5)	20.46 (13.74)	36.25 (32.82)	42.68 (36.78)
104	Joacă	Legitim	Flori	Copil	55.56	0.56 (0.5)	15.95 (10.15)	25.22 (20.61)	33.21 (24.28)
105	Mării	Căpşuni	Şampanie	Spuma	61.9	0.62 (0.49)	19.56 (22.25)	26.19 (27.99)	35.86 (32.95)
106	Scris	Măsurat	Muzical	Instrument	28.57	0.29 (0.46)	13.92 (7.12)	24.19 (27.05)	27.91 (30.44)
107	Înot	Salvare	Pupăză	Colac	74.6	0.75 (0.44)	16.48 (14.07)	22.59 (22.97)	34.22 (31.39)
108	Bătrâni	Renovată	Amanet	Casă	95.24	0.95 (0.21)	9.11 (5.34)	9.37 (6.3)	15.26 (18.08)
109	Centrală	Legume	Neagră	Piaţă	41.27	0.41 (0.5)	19.12 (13.9)	30.6 (29.52)	38.17 (34.46)
110	Timp	Termopan	Evacuare	Fereastră	28.57	0.29 (0.46)	19.12 (11.35)	37.75 (38.73)	42.02 (41.52)
111	Pescărească	Lua	Volei	Plasă	60.32	0.6 (0.49)	13.28 (10.2)	19.84 (21.23)	25.81 (24.47)

Concluded

Appendix 2 - Partial translation of query items from Romanian to English

No.	Query (RO)	Answer (RO)	Query Translation attempt (ENG)	Answer (ENG)
1	Francez Vânătoare Ciocolată	Corn	FRENCH HUNT(ING) CHOCOLATE	HORN [4]
2	Principală Măturător Lumină	Stradă	MAIN SWEEPER LIGHT	STREET
3	Companie Vânătoare Nevăzători	Câine	COMPANY HUNT BLIND	DOG[5]
4	Munte Lance Ascuţit	Vârf	MOUNTAIN SPEAR SHARP	TOP[6]
5	Rază Floare Rupt	Soare	RAY FLOWER BROKEN	SUN[7]
6	Întrebare Circulaţie Zodiacal	Semn	QUESTION TRAFFIC ZODIAC	SIGN[8]
7	Cerneală Rezervor Peniţă	Stilou	INK TANK (RESERVOIR) NIB	PEN
8	Sârmă Vârfuri Înainte	Mers	WIRE TIPS BEFORE	WALKING[9]
9	Corali Trecere Socială	Barieră	CORALS CROSSING SOCIAL	BARRIER
10	Pretext Acoperire Egida	Sub	PRETEXT COVER CARE/AEGIS	UNDER
11	Replică Ploaie Băutură	Acidă	REPLY RAIN DRINK	ACID[10]
12	Şoareci Întrebare Automobil	Capcană	MICE QUESTION CAR	TRAP
13	Urgenţă Telefonic Curte	Apel	EMERGENCY (by)PHONE COURT	APPEAL/CALL
14	Vizual Tragere Semantic	Câmp	VISUAL SHOOTING SEMANTIC	PLAIN/FIELD
15	Mandat Oficiu Timbru	Poştal	MANDATE OFFICE STAMP	POSTAL/POST
16	Scris Cusut Ulei	Maşină	WRITING SEWING OIL	CAR/MACHINE
17	Noapte Rasă Zăpadă	Albă	NIGHT RACE SNOW	WHITE
18	Zâmbet Cântă Document	Fals	SMILE SING DOCUMENT	FALSE/FAKE [11]
19	Navală Sportivă Date	Bază	NAVAL (of)SPORT DATA	BASE
20	Faţă Tenis Moleculară	Masă	CLOTH TENNIS MOLECULAR	TABLE/MASS
21	Franceză Poziţie Cuvânt	Cheie	FRENCH POSITION WORD	KEY
22	Perete Ultimul Electronic	Ceas	WALL LAST ELECTRONIC	WATCH/CLOCK
23	Damă Pantaloni Transmisie	Curea	WOMAN TROUSERS TRANSMISSION	BELT/STRAP
24	Tun Vită Vie	Carne	GUN COW ALIVE	MEAT [12]
25	Pauză Boabe Filtru	Cafea	PAUSE BEANS FILTER	COFFEE
26	Verde Orientală Fructe	Salată	GREEN ORIENTAL FRUITS	SALAD
27	Perete Miez Cocos	Nucă	WALL CORE COCOA	NUT
28	Fân Râu Muncă	Braţ	HAY BAD WORK	ARM [13]
29	Dublă Video Obscură	Cameră	DOUBLE VIDEO OBSCURE	ROOM/CHAMBER[14]
30	Natalităţii Schimb Bancară	Rată	BIRTH EXCHANGE BANK	RATE
31	Ultima Actuală Fixă	Oră	LAST ACTUAL FIXED	HOUR
32	Rinichi Filosofală Seacă	Piatră	KIDNEYS PHILOSOPHER DRY	STONE[15]
33	Pantofi Dansatori Fără	Pereche	SHOES DANCERS WITHOUT	PAIR
34	Legitimă Avocat Cheltuieli	Apărare	LEGITIMATE LAWYER EXPENSES	DEFENSE
35	Ajutor Lucru Dibace	Mână	HELP WORK SKILLED	HAND
36	Gata Doi Gheaţă	Bani	READY TWO ICE	MONEY[16]

Continued on next page

[4] A chocolate horn is a particular sweet in Romania

[5] The third expression refers to a companion dog for the visually impaired

[6] The compounds in English would be MOUNTAIN PEAK, SPEAR HEAD, SHARP TIP

[7] Broken from the sun means very beautiful in Romanian.

[8] In English you would rather say *question mark* than *question sign.*

[9] Walking on tips refers to tiptoeing.

[10] Acid reply means caustic reply or comment.

[11] Singing falsely means to sing out of tune

[12] *Carne de tun* in Romanian refers to soldiers that were thrown into a hopeless battle. *Carne vie* more closely translates to *living flesh* rather than *alive meat.*

[13] An *arm of hay* can act as a measurement for hay

[14] In Romanian *Camera* is room, chamber and camera.

[15] Getting something *out of a dry stone* in Romanian refers to attempting to succeed with very low resources.

[16] In Romanian, someone who is of *ready money* is a son of rich parents which edulges in many extravagant things with the money from their parents; *two money* is the equivalent of two cents, something very cheap; *ice money* is simply cash.

Continued from previous page

No.	Query (RO)	Answer (RO)	Query Translation attempt (ENG)	Answer (ENG)
37	Ou Pisică Picături	Ochi	EGG CAT DROPS	EYE[17]
38	Cal Muncă Economică	Putere	HORSE WORK ECONOMIC	POWER
39	Electronică Prioritară Romană	Poştă	ELECTRONIC PRIORITY ROMANIAN	POST/MAIL
40	Iarbă Păr Telefon	Fir	GRASS HAIR PHONE	THREAD/WIRE[18]
41	Sănătate Credit Identitate	Card	HEALTH CREDIT IDENTITY	CARD
42	Biliard Chibrit Pescuit	Băţ	BILLIARD MATCH FISHING	STICK
43	Mac Vedea Fier	Roşu	POPPY SEE IRON	RED
44	Marinăresc Gordian Cravată	Nod	SAILOR's GORDIAN TIE	KNOT
45	Mese Bal Aşteptare	Sala	TABLES BALL WAITING	HALL/ROOM
46	Practic Şaselea Bun	Simţ	PRACTICAL SIXTH GOOD	SENSE
47	Interzis Dublu Figurat	Sens	FORBIDDEN DOUBLE FIGURATIVE	MEANING
48	Socru Capul Literă	Mare	FATHER-IN-LAW HEAD LETTER	BIG[19]
49	Argint Invitaţie Alai	Nuntă	SILVER INVITATION POMP/RETINUE	WEDDING
50	Astral Diplomatic Delict	Corp	ASTRAL DIPLOMATIC OFFENCE	BODY/CORPS
51	Drum Timp Circuit	Scurt	ROAD TIME CIRCUIT	SHORT
52	Sânge Turcească Abur	Baie	BLOOD TURKISH STEAM	BATH
53	Secret Rutier Civil	Cod	SECRET ROAD/TRAFFIC CIVIL	CODE
54	Rulantă Adezivă Magnetofon	Bandă	ROLLING ADHESIVE RECORDER	TAPE
55	Ziuă Botez Familie	Nume	DAY BAPTISM FAMILY	NAME
56	Oratorică Culinară Contemporană	Artă	ORATORY CULINARY CONTEMPORARY	ART
57	Unghii Pantofi Glaciar	Lac	NAILS SHOES GLACIAR	POLISH/LAKE
58	Ski Biciclişti Aterizare	Pistă	SKI CYCLISTS LANDING	SLOPE/TRACK/STRIP
59	Uşa Pantof Cui	Toc	DOOR SHOE NAIL	HEEL [20]
60	Zăcamânt Nisipuri Medalie	Aur	DEPOSIT SANDS MEDAL	GOLD
61	Corporală Capitală Crimă	Pedeapsă	BODILY CAPITAL MURDER	PUNISHMENT
62	Greşit Primul Dans	Pas	WRONG FIRST DANCE	STEP
63	Miazăzi Briză Vorbă	Vânt	SOUTH BREEZE WORD	WIND[21]
64	Electric Cont Literar	Curent	ELECTRIC ACCOUNT LITERARY	CURRENT
65	Harta Turul Capătul	Lumii	MAP TOUR END	WORLD
66	Piept Pernă Vrabie	Pui	BREAST PILLOW SPARROW	(BABY) CHICKEN
67	Ţine Proaspătă Tăiată	Respiraţia	HOLD FRESH CUT	BREATH
68	Producţie Transport Exprimare	Mijloc	PRODUCTION TRANSPORTATION EXPRESSION	MIDDLE/MEANS
69	Prima Platonică Adevărată	Dragoste	FIRST PLATONIC TRUE	LOVE
70	Gaz Informaţii Canal	Scurgere	GAS INFORMATION CANAL	LEAK[22].
71	Limba Iubire Linie	Maternă	TOUNGUE LOVE LINE	MATERNAL/MOTHER
72	Puşcă Lapte Stele	Praf	GUN MILK STARS	DUST/POWDER
73	Scris Lucru Spălat	Mână	WRITING WORK WASHED	HAND
74	Maximă Ideală Centru	Greutate	MAXIMUM IDEAL MIDDLE	WEIGHT[23]
75	Control Şah Fildeş	Turn	CONTROL CHESS IVORY	TOWER
76	Lumină Bisect Calendaristic	An	LIGHT LEAP (of)CALENDAR	YEAR
77	Tele Călătorie Bord	Jurnal	TELE TRAVEL BOARD	JOURNAL[24]
78	Şarpe Neagră Ureche	Gaură	SNAKE BLACK EAR	HOLE
79	Agricolă Generală Minister	Cultură	AGRICULTURAL GENERAL MINISTRY	CULTURE
80	Forţă Noapte Neagră	Cămaşă	POWER NIGHT BLACK	SHIRT[25]
81	Pescuit Salvare Pneumatică	Barcă	FISHING SALVATION PNEUMATIC	BOAT
82	Rotile Electric Birou	Scaun	WHEEL ELECTRIC DESK	CHAIR
83	Lună Albine Mană	Miere	MOON BEES FOREST[26]	HONEY

Continued on next page

[17] An *eye egg* is a fried egg in Romanian, because it apparently looks like an eye.

[18] In English you wouldn't say *hair thread*, but only hair.

[19] The equivalent of *big letter* is *capital letter*

[20] *Toc usa* means *door casing*; textitToc cui means *stiletto.*

[21] *Vorba in vant* means empty words.

[22] *Canal de scurgere* is a *drain*

[23] The *centre of weight*is *centre of gravity.*

[24] *Telejurnal* is a news bulletin, a *travel journal* is a *travel log*; the *journal on board* is *captain's log.*

[25] *Camasa de forta* is a *straitjacket.*

[26] Inexact translation - *Mana de miere* does translate to *Forest honey*, but the word for *Mana* is not the one for *forest.*

Continued from previous page

No.	Query (RO)	Answer (RO)	Query Translation attempt (ENG)	Answer (ENG)
84	Gură Minerală Izvor	Apă	MOUTH MINERAL SPRING	WATER[27]
85	Tropicală Protejată Amazoniană	Pădure	TROPICAL PROTECTED AMAZONIAN	FOREST
86	Hoţi Rulantă Circulaţie	Bandă	THIEVES ROLLING TRAFFIC	BAND[28]
87	Artificii Armă Lemne	Foc	ARTIFICE WEAPON WOODS	FIRE[29]
88	Ac Centură Naţională	Siguranţă	NEEDLE BELT NATIONAL	SAFETY[30]
89	Muzicală Direcţie Bijuterii	Cutie	MUSICAL DIRECTION/STEERING JEWELRY	BOX
90	Justiţie Şcolii Constituţională	Curtea	JUSTICE (of)SCHOOL CONSTITUTIONAL	YARD/COURT
91	Loc Drept Factor	Comun	PLACE RIGHT FACTOR	COMMON
92	Vizită Bucate Joc	Carte	VISIT FOOD GAME	BOOK/CARD
93	Cont Organe Naţională	Bancă	ACCOUNT ORGANS NATIONAL	BANK
94	Metil Voroneţ Cer	Albastru	METHYL VORONET SKY	BLUE[31]
95	Rufe Vase Clăbuci	Săpun	LAUNDRY DISHES LATHER	SOAP
96	Comercial Vânzare Cosmic	Spaţiu	COMERCIAL SALE COSMIC	SPACE
97	Dormit Plastic Gunoi	Sac	SLEEPING PLASTIC GARBAGE	BAG
98	Adevăr Măr Lumină	Sâmbure	TRUTH APPLE LIGHT	STONE[32]
99	Spălat Tuns Cusut	Maşină	WASHING HAIRCUT SEWING	MACHINE
100	Categorie Autobuz Concert	Bilet	CATEGORY BUS CONCERT	TICKET
101	Proprietate Viaţă Întreprindere	Privată	PROPERTY LIFE ENTERPRISE	PRIVATE
102	Socială Elevi Muncitoare	Clasă	SOCIAL PUPILS WORKER	CLASS
103	Geam Inculpat Drum	Închis	WINDOW DEFENDANT ROAD	CLOSED
104	Joacă Legitim Flori	Copil	PLAY LEGITIMATE FLOWER	CHILD[33]
105	Mării Căpşuni Şampanie	Spuma	SEA STRAWBERRY CHAMPAGNE	FOAM
106	Scris Măsurat Muzical	Instrument	WRITING MEASURING MUSICAL	INSTRUMENT
107	Înot Salvare Pupăză	Colac	SWIMMING SALVATION HOOPOE	LIFE-BUOY[34]
108	Bătrâni Renovată Amanet	Casă	ELDERLY RENOVATED PAWN	HOUSE
109	Centrală Legume Neagră	Piaţă	CENTRAL VEGETABLE BLACK	MARKET
110	Timp Termopan Evacuare	Fereastră	TIME DOUBLE-GLAZING EVACUATION	WINDOW
111	Pescărească Lua Volei	Plasă	FISHING TAKE VOLEY	NET [35]

Concluded

When the answer was translated as two words, one of the words better matches compounds with part of the query words in English, and the other word matches compound(s) with the remaining query words in English.

[27] *Mouth water* does stand in for *moutwash* in Romanian.
[28] A *rolling band* is a *conveyor belt.*
[29] *Fire weapon* is actually *firearm.*
[30] *Safery needle* is a *safety pin.*
[31] *Voronet* is a monastery name in Romania, where famous shade of blue is used.
[32] *stone of truth* is a *grain of truth.*
[33] A *child from flowers* is an *illegitimate child.*
[34] *Colac peste pupaza* means an unpleasant event which happens unexpectedly just after another one.
[35] To *take the net* means to be swindled.
